# Granulocyte colony-stimulating factor (G-CSF) positive effects on muscle fiber degeneration and gait recovery after nerve lesion in MDX mice

**DOI:** 10.1002/brb3.250

**Published:** 2014-08-05

**Authors:** Gustavo F Simões, Suzana U Benitez, Alexandre L R Oliveira

**Affiliations:** Departament of Structural and Functional Biology, Institute of Biology, University of Campinas (UNICAMP)CP 6109, CEP 13083-907, Campinas, SP, Brazil

**Keywords:** Axotomy, G-CSF, muscular dystrophy, Schwann cell

## Abstract

**Background:**

G-CSF has been shown to decrease inflammatory processes and to act positively on the process of peripheral nerve regeneration during the course of muscular dystrophy.

**Aims:**

The aims of this study were to investigate the effects of treatment of G-CSF during sciatic nerve regeneration and histological analysis in the soleus muscle in MDX mice.

**Methods:**

Six-week-old male MDX mice underwent left sciatic nerve crush and were G-CSF treated at 7 days prior to and 21 days after crush. Ten and twenty-one days after surgery, the mice were euthanized, and the sciatic nerves were processed for immunohistochemistry (anti-p75^NTR^ and anti-neurofilament) and transmission electron microscopy. The soleus muscles were dissected out and processed for H&E staining and subsequent morphologic analysis. Motor function analyses were performed at 7 days prior to and 21 days after sciatic crush using the CatWalk system and the sciatic nerve index.

**Results:**

Both groups treated with G-CSF showed increased p75^NTR^ and neurofilament expression after sciatic crush. G-CSF treatment decreased the number of degenerated and regenerated muscle fibers, thereby increasing the number of normal muscle fibers.

**Conclusions:**

The reduction in p75^NTR^ and neurofilament indicates a decreased regenerative capacity in MDX mice following a lesion to a peripheral nerve. The reduction in motor function in the crushed group compared with the control groups may reflect the cycles of muscle degeneration/regeneration that occur postnatally. Thus, G-CSF treatment increases motor function in MDX mice. Nevertheless, the decrease in baseline motor function in these mice is not reversed completely by G-CSF.

## Introduction

The existence of animal models that mimic neuromuscular conditions where the muscular system is changed provides the opportunity to study alterations in the nervous system microenvironment and the possibility to investigate drug actions to reestablish homeostasis in both systems. These studies open paths for improving therapeutic strategies for human patients.

The MDX mouse is the most commonly used mouse strain for Duchenne Muscular Dystrophy (DMD) research. DMD characterized by the absence of dystrophin, is a severe disease that results in damage to muscle fibers including necrosis and regeneration/degeneration and their replacement by connective tissue (Deconinck and Dan 2007). The MDX mouse shows structural abnormalities of the neuromuscular junction including postsynaptic acetylcholine receptor (AChR) declustering, loss of postsynaptic junction folds, increased presynaptic nerve terminal complexity, and muscle denervation (Nagel et al. 1990; Lyons and Slater 1991). Recently, we demonstrated alterations in the muscle interface with the nervous system caused by chronic muscular degeneration processes that retrogradely affected the spinal cord microenvironment, specifically the alpha motoneurons (Simões and Oliveira 2010) and that also caused deficits in peripheral nerve regeneration during the course of DMD (Simões and Oliveira 2012a,b).

In an additional study, we demonstrated that granulocyte colony-stimulating factor (G-CSF) is a potential treatment that can reestablish homeostasis in the spinal cord microenvironment of MDX mice (Simões and Oliveira 2012a,b). Therapeutic use of granulocyte colony-stimulating factor (G-CSF) has been reported for some tissues, such as skeletal muscle, spinal cord, and peripheral nerve (Mirski et al. 2003; Stratos et al. 2007; Pitzer et al. 2008; Hara et al. 2011; Pollari et al. 2011; Simões and Oliveira 2012a,b). G-CSF was initially identified as a hematopoietic cytokine and has been used in both basic research studies and in the clinic for the mobilization of hematopoietic stem cells (Welte et al. 1996; Demetri and Griffin 1999; Metcalf 2008). Recent studies have suggested that G-CSF also plays roles in cell differentiation, proliferation, and survival (Avalos 1996; Harada et al. 2005; Zaruba et al. 2009). The administration of granulocyte colony-stimulating factor (G-CSF) is known to mobilize hematopoietic stem cells from the bone marrow into the peripheral blood (Demetri and Griffin 1999) and is used to treat neutropenia after cytostatic therapy. G-CSF has a wide variety of actions; it reduces apoptosis, drives neurogenesis and angiogenesis, and attenuates inflammation (Boneberg et al. 2000; Saito et al. 2002; Shyu et al. 2004; Lee et al. 2005; Schneider et al. 2005; Kawada et al. 2006).

To date, no study has used G-CSF to compare its effects on the musculoskeletal and neural microenvironment and recovery of motor function after peripheral nerve injury in MDX mice. Thus, we used sciatic nerve axotomy, which induces muscle degeneration along the muscle fibers due to the acute effects of the peripheral nerve injury, followed by treatment with G-CSF. We also used G-CSF to analyze its effects on the neural microenvironment and functional recovery after sciatic nerve crush. This type of axonal injury is followed by Wallerian degeneration (WD) distal to the lesion site (Waller 1850). Cytokines—the mediator molecules of inflammation—are produced during WD and regulate molecular and cellular events including the recruitment and activation of macrophages, activation of Schwann cells, myelin phagocytosis, NGF production, and neuropathic pain (Stoll and Muller 1999). In recent decades, knowledge of the cellular and molecular requirements for peripheral nerve regeneration has increased greatly (Chen et al. 2007). Similarly, many experimental repair therapies have been tested for efficacy on peripheral axon regeneration (Belkas et al. 2004; Bozkurt et al. 2007) and, ultimately, functional recovery (Meek et al. 1999; Varejão et al. 2004; Pfister et al. 2007; Vleggeert-Lankamp 2007; Wang et al. 2008). The use of tests that are sufficiently sensitive to detect functional recovery is indispensable for the assessment of the functionality of axon regeneration. de Medinaceli et al. (1982) established the sciatic function index (SFI), which provides a good indication of the functional contribution of the sciatic nerve to animal gait. A novel automated gait analysis system, the CatWalk, has the advantage of enabling the control of speed of locomotion and enabling automated data acquisition. Moreover, the CatWalk has been shown to be of value in the assessment of static and dynamic gait parameters in a variety of central and peripheral nerve injury models (Hamers et al. 2006; Deumens et al. 2007).

## Material and Methods

### Animals

C57BL/10 and MDX male mice were obtained from the Multidisciplinary Center for Biological Research (CEMIB), State University of Campinas, at 6 weeks of age. The mice were grouped and acclimatized in cages for 1 week with ad libitum access to food and water under controlled light (light/dark cycle of 12 h) and temperature conditions (21°C) in racks specific for mice at the Laboratory of Nerve Regeneration, Department Structural and Functional Biology/Unicamp, Brazil. The study was approved by the Institutional Committee for Ethics in Animal Experimentation (CEEA/IB/UNICAMP, proc. 1776-1) and the experiments were performed in accordance with the guidelines of the Brazilian College for Animal Experimentation (CONCEA).

The experimental groups are presented in Table 1 and Table 2. In the drug-treated groups, the mice were pretreated with 200 mg/kg/day G-CSF subcutaneously for 7 days prior to and 10 days after the sciatic nerve crushing. In the placebo groups, the mice were pretreated with 200 *μ*L of 5% sucrose. From the 11th to the 21st day, G-CSF was administered on alternate days.

**Table 1:** Groups and techniques used in mice subjected to sciatic nerve crushing

		Immunohistochemistry		Electron Microscopy		Functional Recovery		H&E Muscle	
					
Group	Experiment - 21 d.p.o	C57BL/10	MDX	C57BL/10	MDX	C57BL/10	MDX	C57BL/10	MDX
1	Nonlesioned untreated	5	5	5	5	5	5	–	–
2	Nonlesioned + G-CSF	5	5	5	5	5	5	–	–
3	Axotomized untreated	–	–	–	–	–	–	5	5
4	Axotomized + G-CSF	–	–	–	–	–	–	5	5
5	Crushed untreated	5	5	5	5	5	5	–	–
6	Crushed + placebo	5	5	5	5	5	5	–	–
7	Crushed + G-CSF	5	5	5	5	5	5	–	–

**Table 2:** Groups and techniques used in mice subjected to sciatic nerve transection

		Functional Recovery		H&E Muscle	
			
Group	Experiment - 10 d.p.o	C57BL/10	MDX	C57BL/10	MDX
1	Nonlesioned untreated	5	5	–	–
2	Nonlesioned + G-CSF	5	5	–	–
3	Axotomized untreated	–	–	5	5
4	Axotomized + G-CSF	–	–	5	5
5	Crushed untreated	5	5	–	–
6	Crushed + placebo	5	5	–	–
7	Crushed + G-CSF	5	5	–	–

### Immunohistochemistry

For immunohistochemistry, the right and left sciatic nerves were frozen and embedded in Tissue-Tek (Miles Inc., Torrance, CA) and frozen in liquid nitrogen at −24°C for cryostat longitudinal sectioning (12 *μ*m). Primary mouse anti-neurofilament (1:100; Chemicon, Billerica, MA), goat anti-p75^NTR^ (1:200; Santa Cruz, Dallas, TX), and anti-sera were used. The anti-sera were diluted in BSA and Triton X-100 in 0.01 mol/L PBS. The sections were incubated overnight at 4°C in a moist chamber. After rinsing in 0.01 mol/L PBS, the sections were incubated with Cy3- or Cy2-labeled secondary anti-sera (1:250; Jackson Immunoresearch, West Grove, PA) for 45 min in a moist chamber at room temperature. The sections were then rinsed in PBS, mounted in a mixture of glycerol/PBS (3:1), and observed using a Nikon TS100 microscope that was equipped with a digital camera (Nikon, Tokyo, Japan, DXM1200i).

Three alternate sections were chosen from each animal (*n* = 5 for each group). These sections were used to capture images at a final magnification of 6200× with all settings unchanged. Quantification was performed using the enhance contrast and density slicing feature of the ImageJ software (version 1.33u; National Institutes of Health, Bethesda, MD). The integrated pixel density was measured at the distal nerve region relative to lesion area. The data are shown as the mean ± standard error of the mean (SE).

### Sciatic nerve lesion

The mice were anesthetized using a mixture of Vetaset (ketamine; Fort Dodge, IA, 50 mg/kg) and kensol (xylazine; Körnig, Argentina, 10 mg/kg), totaling 0.12 mL/25 g of body weight. After trichotomy of the left mid-thigh, a skin incision of approximately 1.5 cm was made using a scalpel. The skin and the thigh muscles were carefully retracted, thereby exposing the sciatic nerve, which was injured at the level of the obturator foramen. The left nerve crush was performed using a clamp number 4 with constant pressure for 10 sec for all mice (Xin et al. 1990). A surgical knot was tied in the muscle tissue adjacent to the lesion site to mark the location where the nerve was crushed.

For morphological analysis of the soleus muscle, total sciatic nerve axotomy was performed using a micro scissors, and a segment of 3 mm of the distal nerve stump was removed and diverted from its normal direction to avoid realignment between the stumps.

### Electron microscopy

The nerves were dissected out and stored overnight in fixative (1.5% paraformaldehyde, 2.5% glutaraldehyde in 0.1 mol/L PB, pH 7.34) at 4°C. The specimens were then trimmed, osmicated, dehydrated, and embedded in Durcupan (Fluka, Steinheim, Switzerland). Ultrathin sections from the L4-L6 segments and nerves were placed on formvar-coated copper grids, contrasted with uranyl acetate and lead citrate and examined using a Tecnai Biotwin G2 Spirit transmission electron microscope (FEI Company, Eindhoven, the Netherlands), which was operated at 120 kV. An area equivalent to 30% of the total area of the nerve was selected and sequential photomicrographs were taken and assembled together for nerve fiber quantification. The following parameters were considered for quantification: number of myelinated axons in normal nerves, number of myelinated axons in regenerated nerves, number of degenerating myelinated axons (using the program Adobe Photoshop 5.0; San Jose, CA). Diameter of myelinated fibers (DMF), which corresponds to the diameter containing the axon and its myelin sheath (Figure S1A); diameter of myelinated axons (DMA), corresponding to the diameter of the axon without myelin sheath (Figure S1B), myelin thickness (MT), calculated from the difference between the diameter of the fibers and their respective axons divided by 2 (Figure S1C), and the “g” ratio (GR), obtained from the ratio DMA/DMF.

The diameter of the fibers and myelinated axons were obtained from the values of their respective perimeters (P) and calculated using the formula D = P/*π*. The difference between the diameter of the myelinated fibers [DMF] and the diameter of the myelinated axons [DMA] provided the thickness of the myelin sheath [EBM] (Mayhew and Sharma 1984). G ratio corresponds to the quotient of DMA and DMF, and is a parameter that expresses functional nerve regeneration (Smith and Koles 1970).

### Functional recovery of the sciatic nerve

Initially, a functional assessment with normal mice was performed using the “Walking Track Test” (Catwalk System, Wageningem, the Netherlands) to obtain a normal gait. The mice were subjected to functional assessments performed daily over the first 10 days after injury and then every other day until day 21 after sciatic nerve crush. Measurements were made based on the sciatic nerve motor recovery index. The measurements were performed based on two parameters: the distance between the first and fifth toes (toe spread, TS) and the distance between the third toe and the heel (print length, PL). These parameters were used to measure the footprints and the values were used in the following formula described by de Medinaceli et al. (1982): SFI = 118.9 (ETS-NTS/NTS) -51.2 (ELP-NLP/NLP) -7.5 (E = injured side, N = normal side).

### Morphological analysis of the soleus muscle fibers

After perfusion and fixation in 4% formalin, the muscles were dissected out and processed for embedding in Paraplast. Sections of 5 mm in thickness were obtained using a rotary microtome, deparaffinized using routine procedures, and stained with hematoxylin and eosin (H&E) for morphological analysis, including a qualitative evaluation of cellular infiltrates (macrophages and polymorphonuclear cells). All muscle fibers were considered for quantification. The fibers were identified as unlesioned, degenerated or regenerated. The unlesioned muscle fibers were identified as those in which no edema was evident and that exhibited the presence of one or more nuclei located in the peripheral region of the muscle cell (Pastoret and Sebille 1995). Degenerated muscle fibers were identified as those showing swelling and inflammatory cell infiltration. Regenerated muscle fibers were identified by the absence of peripheral nuclei (Pastoret and Sebille 1995).

### Statistical analyses

The data are presented as the mean ± SEM and were analyzed using one-way ANOVAs followed by Bonferroni post hoc tests for multiple comparisons at *P* ≤ 0.05 (*), *P* ≤ 0.01 (**) and *P* ≤ 0.001 (***).

## Results

### Immunoreactivity for neurofilament after nerve injury

**Figure 1:**
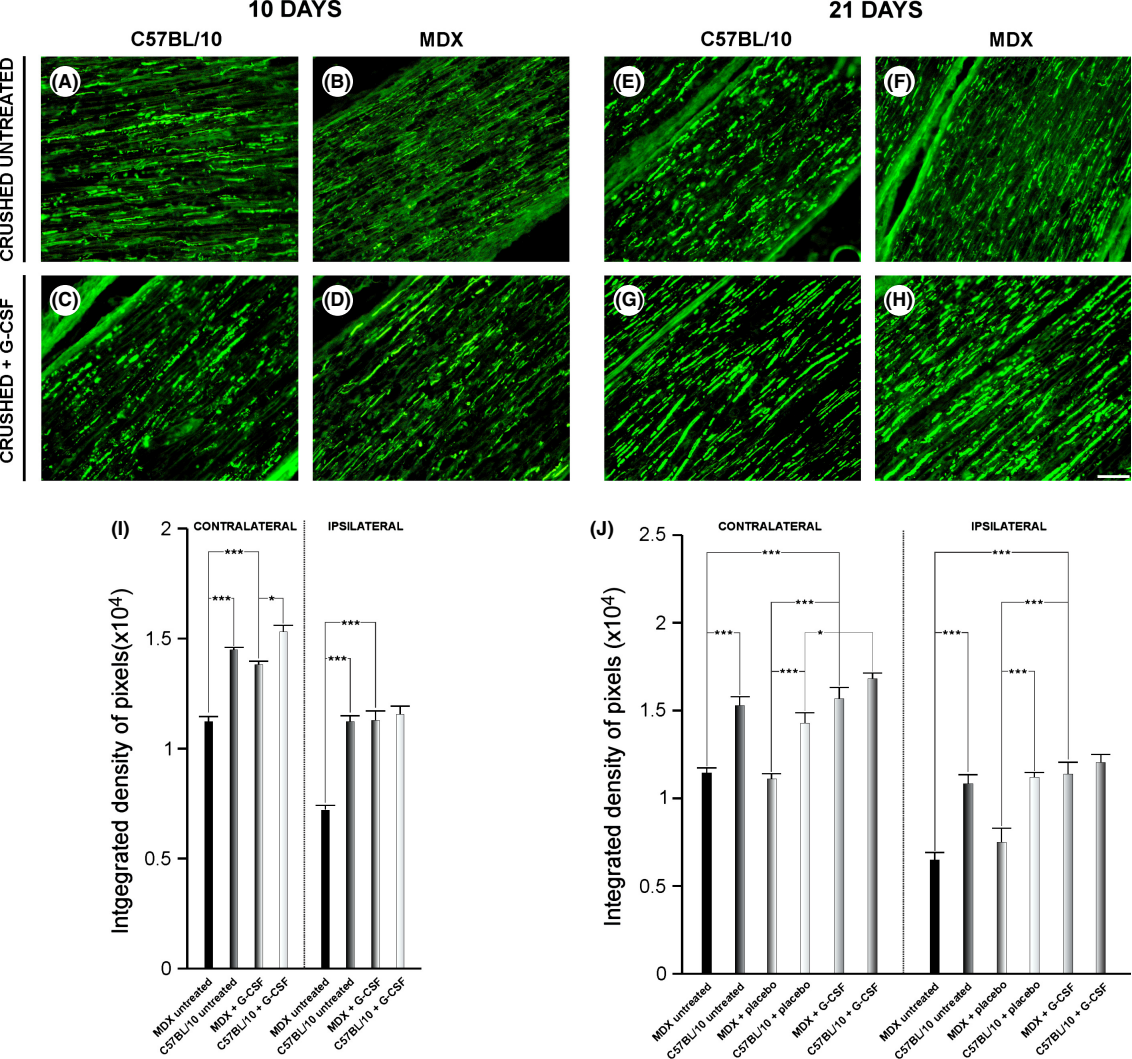
Anti-neurofilament immunostaining at 10 and 21 days after sciatic nerve crush. (A and B) Untreated, ipsilateral nerve 10 days after lesion. (C and D) Ipsilateral nerve + G-CSF 10 days after lesion. (E and F) Untreated, ipsilateral nerve 21 days after lesion. (G and H) Ipsilateral nerve + G-CSF 21 days after lesion. Note that there is a reduced immunoreactivity in MDX mice of all groups. However, G-CSF increased immunoreactivity for neurofilament. (I and J) Quantitative analysis of the pixel integrated density of the immunostaining on each side. *N* = 5 for all experiments. For A–H, the magnification is X200 (scale bar denotes 50 *μ*m). The quantification was performed over the entire nerve area shown in each photo. For I and J, *P ≤ 0.05 and ***P ≤ 0.001 vs. CT, values are the mean ± SEM.

Ten days after sciatic nerve crushing, the quantification of neurofilament immunohistochemistry (integrated density of pixels) demonstrated a significant reduction in MDX untreated compared with C57BL/10 untreated group (23% contralateral and 36% ipsilateral, *P* ≤ 0.001, and *P* ≤ 0.001, respectively, Fig. 1I).

Following G-CSF treatment, an increase in the neurofilament expression in MDX mice was observed (24% contralateral and 57% ipsilateral, *P* ≤ 0.05 and *P* ≤ 0.001, respectively, Fig. 1I). The C57BL/10 + G-CSF group showed no significant improvement following G-CSF treatment (6% contralateral and 3% ipsilateral, *P* > 0.05, and *P* > 0.05, respectively, Fig. 1I).

Twenty-one days after injury, a decrease in immunoreactivity for neurofilament was observed in the untreated and treated in both strains (Fig. 1J). When comparing the untreated and placebo groups with treated groups, the difference was more significant in MDX + G-CSF group, which showed an increase in the expression of neurofilament and in the reorganization of the regenerated fibers (improvement of 37% - contralateral and 76% - ipsilateral, *P* ≤ 0.001 and *P* ≤ 0.001, respectively for the untreated group, and 43% contralateral and 52% ipsilateral, *P* ≤ 0.001 and *P* ≤ 0.001, respectively for the placebo group). In C57BL/10 + G-CSF contralateral group, an increase in immunoreactivity for neurofilament was observed compared to the placebo group (17%, *P* ≤ 0.05, Fig. 1J). In other cases, no significant effect of G-CSF treatment was observed.

### Immunoreactivity for p75^ntr^ after nerve injury

**Figure 2:**
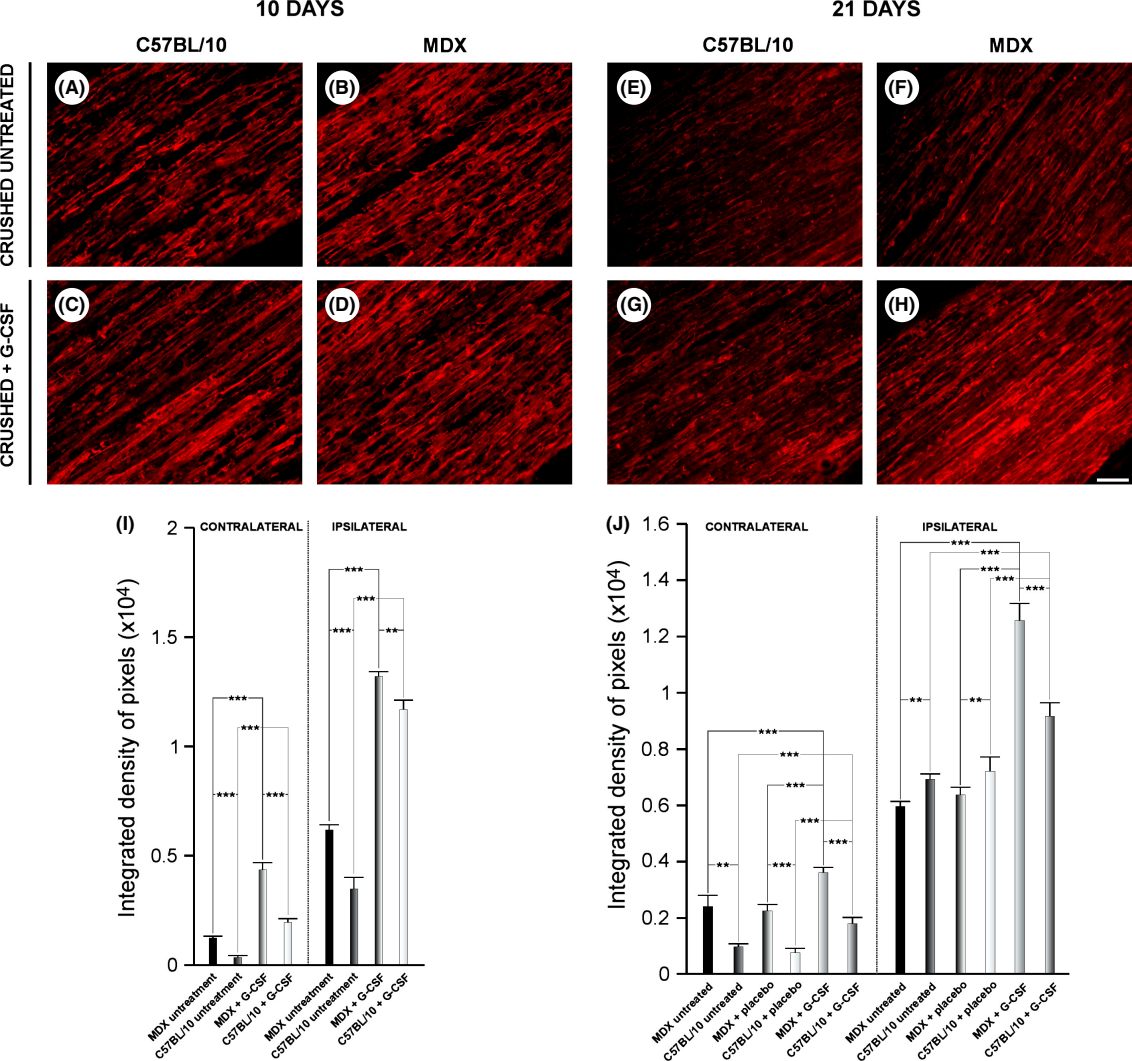
Immunolabelling using anti- p75^NTR^ (low-affinity receptor for neurotrophins) at 10 and 21 days after sciatic nerve crush. (A and B) Untreated, ipsilateral nerve 10 days after lesion. (C and D) Ipsilateral nerve + G-CSF 10 days after lesion. (E and F) Untreated, ipsilateral nerve 21 days after lesion. (G and H) Ipsilateral nerve + G-CSF 21 days after lesion. Note the increase in immunoreactivity in MDX mice compared with C57BL/10 mice for all groups. However, G-CSF increased immunostaining for p75^NTR^ in contralateral and ipsilateral tissues. (I and J) The quantitative analysis of the pixel integrated density of the right and left sides. *N* = 5 for all experiments. For A–H, the magnification is X200 (scale bar denotes 50 *μ*m). The quantification was performed over the entire nerve area shown in each photo. For I and J, *P ≤ 0.05, **P ≤ 0.01, and ***P ≤ 0.001 vs. CT, values are the mean ± SEM.

Ten days after sciatic nerve crush, p75^NTR^ (a low-affinity receptor for neurotrophins) immunohistochemistry revealed a greater increase in untreated MDX mice compared with untreated C57BL/10 mice (Fig. 2).

Following G-CSF treatment, p75^NTR^ expression increased in both strains (MDX mice: ipsilateral, 248%, contralateral, 114%, *P* ≤ 0.001 and *P* ≤ 0.001, respectively; C57BL/10 mice: ipsilateral, 482%, contralateral, 237%, *P* ≤ 0.001 and *P* ≤ 0.001, respectively, Fig. 2I).

At 21 days after sciatic nerve crush, we observed greater reactivity for p75^NTR^ in the untreated, placebo, and treated MDX mice. This increase was most evident when the treated group (50% contralateral and ipsilateral 111%, for comparison between untreated and G-CSF groups, *P* ≤ 0.001 and *P* ≤ 0.001, respectively; 61% contralateral and ipsilateral 110%, for comparison between placebo and G-CSF groups, *P* ≤ 0.001 and *P* ≤ 0.001, respectively, Fig. 2J).

### Morphometry and count of the degenerated fibers

**Figure 3:**
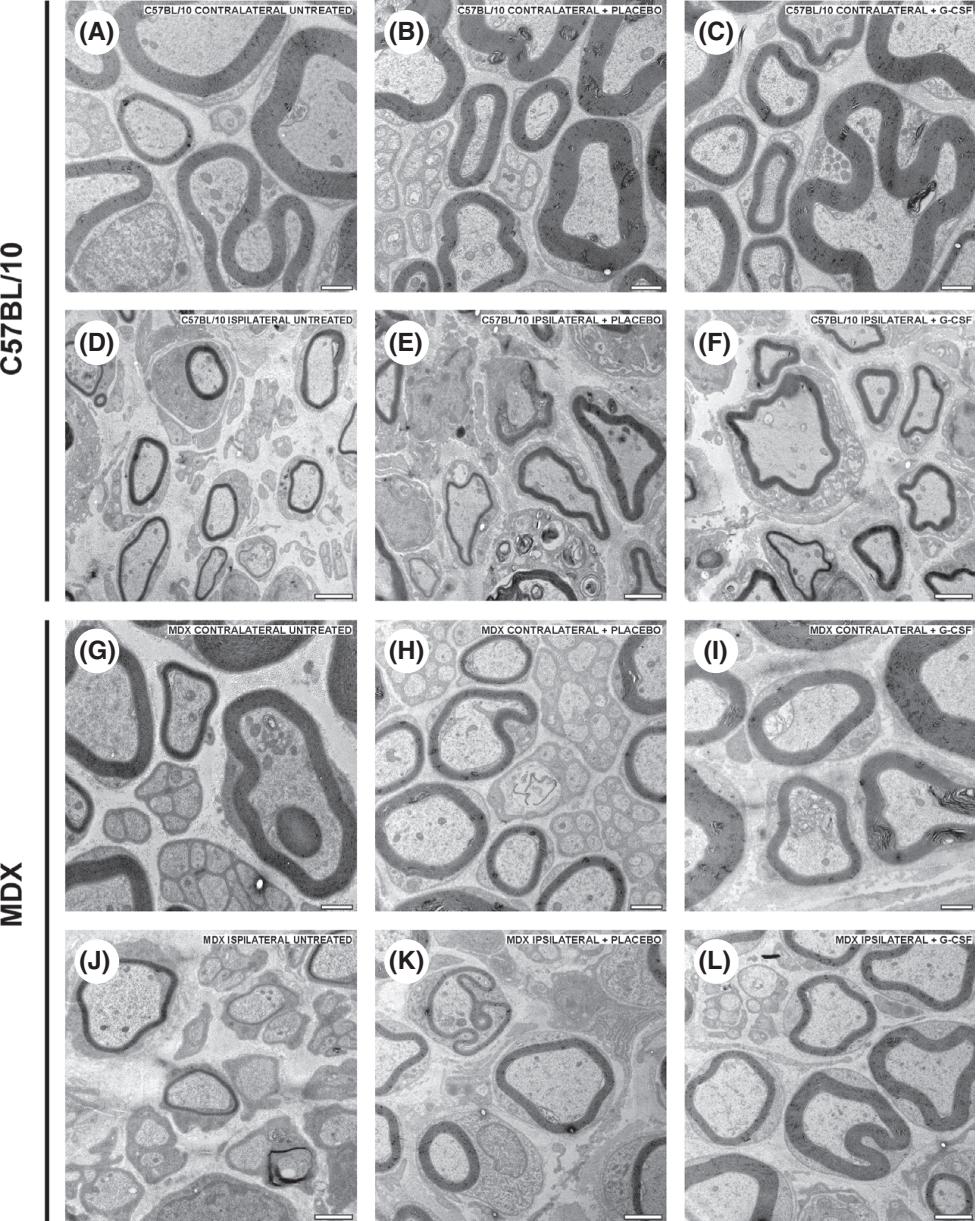
Representative photomicrographs of the sciatic nerve pre- and postlesion in each strain studied. (A–C) Unlesioned nerves (contralateral) of C57BL/10 mice. D–F Lesioned nerves (ipsilateral) of C57BL/10 mice. G–I Unlesioned nerves of MDX mice. J–M Lesioned nerves of MDX mice. Scale bar denotes 1 *μ*m (A–C and G–I) or 2 *μ*m (D–F and J–M).

**Figure 4:**
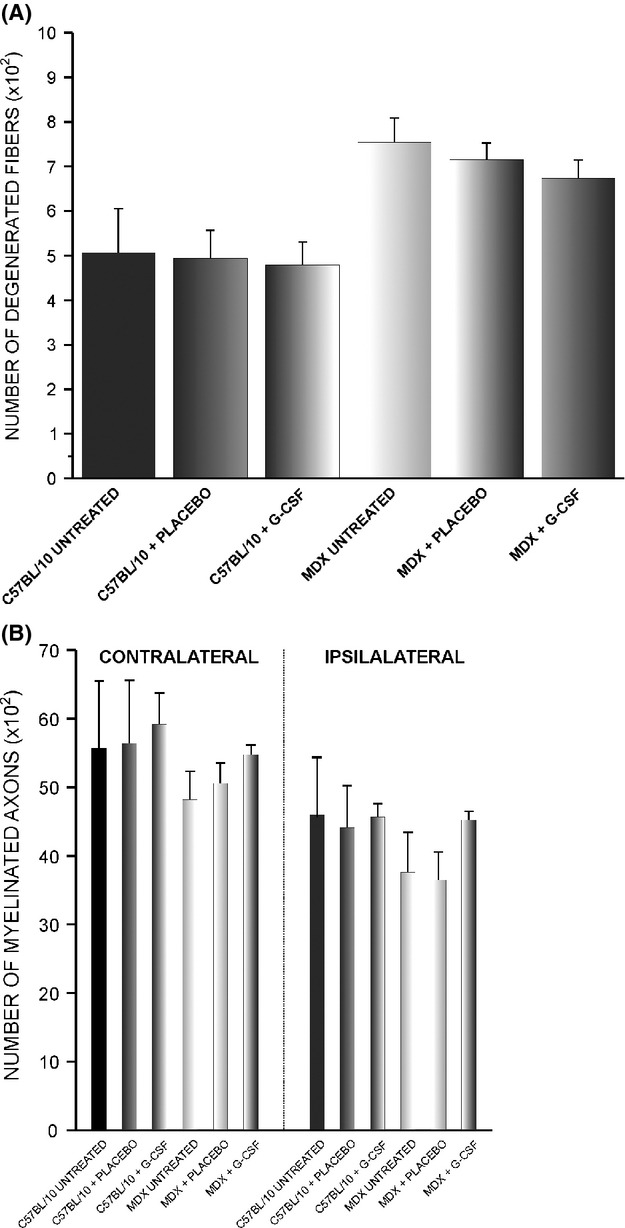
(A) Quantitative analysis of the total number of myelinated axons. (B) Quantitative analysis of the number of degenerated fibers.

Figure 3 shows the ultrastructural images of nerves at 21 days after crush injury. Figure 4A shows the total number of degenerated fibers following nerve injury in the untreated, placebo, and G-CSF-treated groups. We can observe a tendency in the neuroprotection on the nerve fibers after G-CSF treatment in MDX mice. The MDX ipsilateral + G-CSF group showed a reduction of 11% (*P* > 0.05) and 6% (*P* > 0.05) in the number of degenerated fibers compared with untreated and placebo groups, respectively. Similarly, in C57BL/10 ipsilateral + G-CSF group, a reduction of 6% (*P* > 0.05) and 4% (*P* > 0.05) relative to the untreated and placebo groups, respectively, was observed.

### Total number of myelinated axons

Figure 4B shows the total number of myelinated axons following nerve injury in untreated, placebo, and G-CSF-treated groups. In the MDX contralateral + G-CSF group, we observed an increase of 13% (*P* > 0.05) and 9% (*P* > 0.05) over untreated mice and mice treated with placebo, respectively, in the total number of myelinated fiber. Similarly, an increase of approximately 7% (*P* > 0.05) and 5% (*P* > 0.05) relative to untreated and placebo mice, respectively, was observed in C57BL/10 contralateral untreated group. We observed a 13% reduction in the total number of axons in MDX mice compared with C57BL/10.

Ipsilateral analysis revealed that G-CSF treatment increased the number of myelinated axons in MDX mice because an increase of 21% (*P* > 0.05) and 20% (*P* > 0.05) over untreated and placebo mice, respectively, was observed. In C57BL/10 ipsilateral + G-CSF group, we observed an increase of 1% (*P* > 0.05) and 3% (*P* > 0.05) compared with the untreated and placebo groups, respectively.

### Diameter of myelinated fibers

The total myelin fiber quantification in the crushed nerves that were either untreated or treated with G-CSF (Figure S2). When analyzing at contralateral to the lesion in MDX untreated mice, we observed myelin fibers with diameters ranging from 2.16 to 22.44 *μ*m. The highest percentage of fibers was observed in the range of 5.5 to 10.5 *μ*m, representing 72.16% of total fibers. The remaining myelinated fibers (27.83%) were distributed to other classes. In C57BL/10 untreated mice, we observed myelin fibers with diameters ranging between 1.36 and 19.44 *μ*m. The highest percentage of fibers was in the range of 3.5 to 8.5 *μ*m, representing 76.87% of total fibers. The remaining myelinated fibers (23.13%) were distributed to other classes.

During the ipsilateral analysis of MDX untreated group, we observed myelin fibers with diameters ranging from 1.13 to 13.18 *μ*m. The highest percentage of fibers was found in the range of 3.5 to 7.5 *μ*m, corresponding to 83.7% of the total fibers. The remaining myelinated fibers (16.3%) were distributed to other classes. In C57BL/10 ipsilateral untreated group, we observed myelin fibers with diameters ranging from 1.93 to 10.47 *μ*m. The highest percentage of fibers was found in the range of 3.5 to 6.5 *μ*m, representing 85.57% of the total fibers. The remaining myelinated fibers (14.42%) were distributed to other classes.

When analyzing the group MDX contralateral + placebo, we observed myelinated fibers with diameters ranging from 2.16 and 20.66 *μ*m. The highest percentage of fibers was found in the range of 4.5 to 10.5 *μ*m, representing 75.13% of the total fibers. The remaining myelinated fibers (24.86%) were distributed to other classes. In C57BL/10 contralateral + placebo, myelin fibers showed diameters ranging from 2.06 to 18.61 *μ*m. The highest percentage of fibers was found in the range of 5.5 to 10.5 *μ*m, representing 67.54% of the total fibers. The remaining myelinated fibers (32.45%) were distributed to other classes. The analysis of MDX ipsilateral + placebo revealed myelinated fibers with diameters ranging from 2.13 and 12.55 *μ*m. The highest percentage of fibers was found in the range of 4.5 to 7.5 *μ*m, representing 73.77% of the total fibers. The remaining myelinated fibers (26.23%) were distributed to other classes. In C57BL/10 ipsilateral + placebo, we observed myelin fibers with diameters ranging from 2.12 to 12.39 *μ*m. The highest percentage of fibers was found in the range of 4.5 to 7.5 *μ*m, representing 71.12% of the total fibers. The remaining myelinated fibers (28.87%) were distributed to other classes.

Myelin fibers in the MDX contralateral + G-CSF group showed diameters of 2.38 to 17.74 *μ*m, with the highest percentages of fibers in the range of 4.5 to 10.5 *μ*m, corresponding to 74.41% of the total fibers. The remaining myelinated fibers (25.58%) were distributed to other classes. In the C57BL/10 contralateral + G-CSF group, we observed myelin fibers with diameters ranging from 1.43 to 19.27 *μ*m. The highest percentage of fibers was found in the range of 4.5 to 10.5 *μ*m, representing 78.25% of total fibers. The remaining myelinated fibers (21.74%) were distributed to other classes.

Myelin fibers in the MDX ipsilateral + G-CSF group showed diameters of 1.66 to 12.55 *μ*m, with the highest percentages of fibers in the range of 3.5 to 6.5 *μ*m, corresponding to 80.94% of the total fibers. The remaining myelinated fibers (19.05%) were distributed to other classes. In the C57BL/10 ipsilateral + G-CSF group, we observed myelin fibers with diameters ranging from 1.77 to 10.80 *μ*m. The highest percentage of fibers was found in the range of 3.5 and 6.5 *μ*m, representing 89.18% of total fibers. The remaining myelinated fibers (10.81%) were distributed to other classes.

### Diameter of myelinated axons

The diameter of the myelinated axons was measured (Figure S3). In the MDX contralateral untreated group, the axon diameters ranged from 1.11 to 15.64 *μ*m, with most axons in the range of 4.5 to 9.5 *μ*m, representing 71.24% of the total. The remaining 28.76% of axons were distributed to other classes. In C57BL/10 contralateral untreated group, we observed axon diameters ranging from 1.28 to 14.83 *μ*m, with most being between 3.5 and 8.5 *μ*m, corresponding to 81.73% of the total. The remaining, 18.26% of axons were distributed to other classes. In the MDX ipsilateral untreated group, the axon diameters ranged from 1.13 to 11.26 *μ*m with most being between 2.5 and 6.5 *μ*m, corresponding to 90.36% of the total axons. The remaining axons (9.64%) were distributed to other classes. In C57BL/10 ipsilateral untreated group, we observed axons with diameters ranging from 1.01 to 9.31 *μ*m, and the largest percentage of axons were found in the range of 2.5–5.5 *μ*m, corresponding to 90.77% of the total axonal fibers. The remaining axons (9.23%) were distributed to other classes.

In MDX contralateral + placebo group, the axon diameters ranged from 1.10 to 14.24 *μ*m. Most axons ranged from 2.5 to 8.5 *μ*m, corresponding to 91.23% of the total axonal fibers. The remaining axons (8.76%) were distributed to other classes. C57BL/10 contralateral + placebo group showed axon diameters of between 1.25 and 13.09 *μ*m, with most being between 2.5 and 7.5 *μ*m, corresponding to 87.79% of total axons. The remaining axons (12.21%) were distributed to other classes.

The MDX ipsilateral + placebo group showed axons with diameters ranging from 1.00 to 10.07 *μ*m, and most ranged from 2.5 to 5.5 *μ*m, representing 81.29% of the total axonal fibers. The remaining 18.71% of axons were distributed to other classes. C57BL/10 ipsilateral + placebo group showed axon diameters ranging from between 1.14 to 10.87 *μ*m, and most were between 2.5 and 6.5 *μ*m, corresponding to 89.32% of axons. The remaining axons (10.68%) were distributed to other classes.

In the MDX contralateral + G-CSF group, axon diameters ranged from 1.29 to 14.49 *μ*m. The highest percentage of axons was found between 2.5 and 7.5 *μ*m, representing 87.76% of the axons, and the remaining axons (12.24%) were distributed to other classes. In C57BL/10 contralateral + G-CSF group, axon diameters ranged from 1.13 to 13.65 *μ*m. Most diameters were between 2.5 and 7.5 *μ*m, corresponding to 92.91% of total axonal fibers. The remaining axons (7.09%) were distributed to other classes. The axon diameters in the MDX ipsilateral + G-CSF ranged from 1.10 to 10.07 *μ*m, with the largest percentage of axons in the range 2.5–4.5 *μ*m, corresponding to 76.29% of the total axons. The remaining 23.71% of axons were distributed to other classes. In C57BL/10 ipsilateral + G-CSF group, diameters of axons ranged from 1.08 to 29.28 *μ*m, with most found between 2.5 and 4.5 *μ*m, corresponding to 84.9% of total axonal fibers. The remaining axons (15.1%) were distributed to other classes.

### Myelin thickness and the “g” ratio

Morphometric analysis of the thickness of myelin revealed a reduction in both strains when treated with G-CSF compared with untreated groups (Figure S4 and S5, respectively). Myelin thicknesses ranged from 0.37 to 6.1 *μ*m in contralateral from MDX untreated group. Most thicknesses ranged from 1.7 to 2.7 *μ*m, corresponding to 44.79% of total, and the remaining (55.21%) distributed to other classes. The “g” ratio in this group was between 0.43 and 0.92. Myelin thicknesses ranged from 0.44 to 8.8 *μ*m in C57BL/10 untreated group, with the most frequent thicknesses being between 1.9 and 3.3 *μ*m, corresponding to 44.51% of total axons, whereas the remaining axons (55.49%) distributed to other classes. The “g” ratio in this group was between 0.16 and 0.92.

In MDX ipsilateral + G-CSF group, we observed that the myelin thicknesses varied from 0.12 to 3.87 *μ*m, with most thicknesses being between 0.5 and 1.3 *μ*m, corresponding to 76% of the axons, whereas the other thicknesses (24%) were distributed to other classes. The “g” ratio in this group was between 0.50 and 0.99. In C57BL/10 ipsilateral + G-CSF group, myelin thicknesses ranged from 0.14 to 2.63 *μ*m, with most frequency thicknesses ranging from 0.5 to 1.3 *μ*m, representing 87.44% of axons. The remaining 12.56% were distributed to other classes. The “g” ratio in this group was between 0.41 and 0.99.

In the MDX contralateral + placebo group, we observed myelin thicknesses of 0.43 to 5.03 *μ*m, with the greatest proportion occurring between 1.1 and 2.5 *μ*m, corresponding to 49%. The remaining 51% were distributed to other classes. The “g” ratio in this group was between 0.37 and 0.87. In the C57BL/10 contralateral + placebo group, we observed myelin thicknesses ranging from 0.18 to 7.95 *μ*m, with most being between 1.3 and 2.7 *μ*m, corresponding to 49.4% of the total axons. The “g” ratio in this group was between 0.26 and 0.96.

In the MDX ipsilateral + placebo group, the myelin thicknesses were 0.3–3.87 *μ*m with most being between 0.7 and 1.5 *μ*m, representing 73.25% of axons. In this group, the “g” values were between 0.45 and 0.92. In C57BL/10 ipsilateral + placebo group, the myelin thicknesses ranged from 0.41 to 4.7 *μ*m, with most being between 0.9 to 1.9 *μ*m, corresponding to 82.21% of the total axons. The “g” ratio in this group was between 0.39 and 0.91.

Following treatment with G-CSF, we observed an increase in myelin thickness in MDX mice, particularly on the contralateral side. The myelin thicknesses ranged from 0.1 to 8.51 *μ*m, with most being between 0.1 and 8.51 *μ*m, corresponding to 56.86% of all thicknesses. The remaining 43.14% were distributed to other classes. The “g” ratio in this group was between 0.39 and 0.99. Following G-CSF, the myelin thicknesses in C57BL/10 mice ranged from 0.22 to 8.03 *μ*m. Most thicknesses were between 0.9 and 2.9 *μ*m, corresponding to 60.5% of the total axons. The “g” ratio in this group was between 0.21 and 0.97.

In contrast to the contralateral side, myelin thickness decreased in MDX mice treated with G-CSF. Myelin thickness ranged from 0.13 to 3.87 *μ*m, with most thicknesses ranging from 0.5 to 1.3 *μ*m, representing 77.12% of axons. The “g” ratio in this group was between 0.49 and 0.96. The decrease was also observed in C57BL/10 contralateral + G-CSF group, where the myelin thicknesses ranged from 0.24 to 2.75 *μ*m, with most being between 0.5 and 1.3 *μ*m, which corresponded to 81.57% of the total axons. The “g” ratio in this group was between 0.23 and 0.92.

### Analysis of motor function following sciatic nerve crush

**Figure 5:**
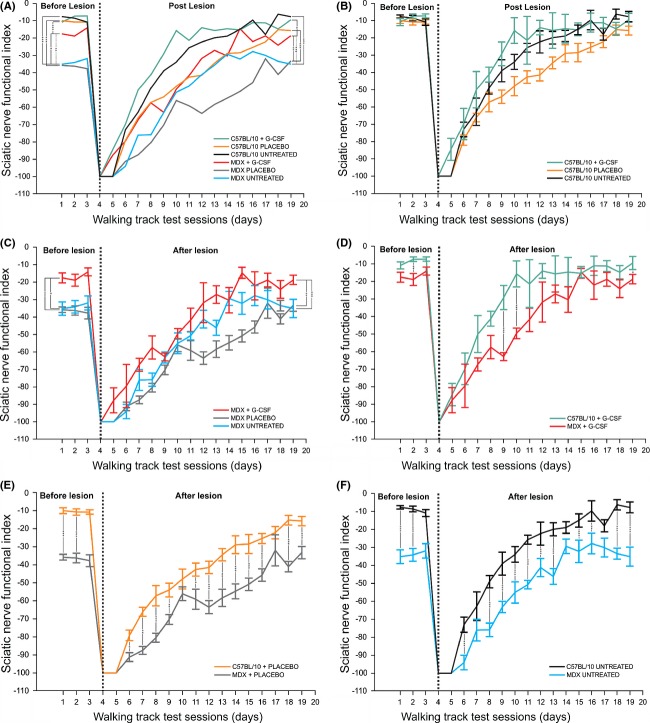
Graphs showing the recovery of motor function in C57BL/10 and MDX strains. (A) Comparison of functional recovery of the sciatic nerve in untreated, placebo, and G-CSF groups in MDX and C57BL/10 strains. (B) Comparison of functional recovery of the sciatic nerve in untreated, placebo, and treated groups of the C57BL/10 strain. (C) Comparison of the functional recovery of the sciatic nerve between untreated, placebo, and treated groups of the MDX strain. (D) Comparison of the functional recovery of the sciatic nerve in MDX and C57BL/10 stains treated with G-CSF. (E) Comparison of the functional recovery of the sciatic nerve in MDX and C57BL/10 stains treated with placebo. (F) Comparison of the functional recovery of the sciatic nerve in the MDX and C57BL/10 stains that were untreated.

Prior to injury, MDX untreated and placebo groups showed motor deficits when compared with same groups in C57BL/10 (MDX untreated −35.14 ± 3.82, mean sciatic function index [SFI] ± SE; C57BL/10 untreated, −7.63 ± 0.94, *P* ≤ 0.001, Fig. 3F; MDX + placebo, −35.82 ± 1.57; C57BL/10 + placebo, −10.01 ± 1.63, *P* ≤ 0.001, Fig. 5E). However, G-CSF treatment improved motor function in MDX mice prior to left sciatic nerve crush, resulting in no statistically significant difference between the MDX and C57BL/10 groups (MDX + G-CSF, −17.58 ± 2.85; C57BL/10 + G-CSF, −10.84 ± 2.00, *P* > 0,05, Fig. 5D). Motor function in MDX + G-CSF mice improved dramatically (∼99.8%) compared with the MDX untreated group. A similar improvement (∼103%) was observed when compared with the placebo group.

In the treated groups, motor function regeneration was more effective at the end of the first 10 days of treatment after injury. The positive effects of the drug on functional regeneration were most evident during the first few days; the treated mice elicited sufficient pressure for the Cat Walk system to capture foot prints at 2 days after the lesion. Thus, an increase in the motor function of 14% in MDX mice treated with G-CSF was observed when they were compared with untreated or placebo mice at 2 days after the lesion. This difference became more evident on the third day after the lesion when the treated group showed an improvement of 18% and 26% over the untreated and placebo groups, respectively. Similarly, in C57BL/10 + G-CSF group at 2 days after injury showed an improvement of 18% over untreated and placebo groups.

After 3 weeks, the curve in motor recovery remained similar between strains; however, MDX mice showed a significant reduction in motor function compared with C57BL/10 mice (untreated group, 379%; placebo group, 112%; G-CSF group, 96%). MDX + G-CSF group showed greater motor function than did untreated MDX mice (∼97%) and the placebo group (∼77%).

### Morphological analysis of the soleus muscle in MDX mice treated with G-CSF

Morphological analysis of soleus muscle showed a trend toward fiber protection following G-CSF treatment in MDX mice. In line with such results, drug treatment reduced macrophage and polymorphonuclear infiltrates, as seen by H&E staining.

**Figure 6:**
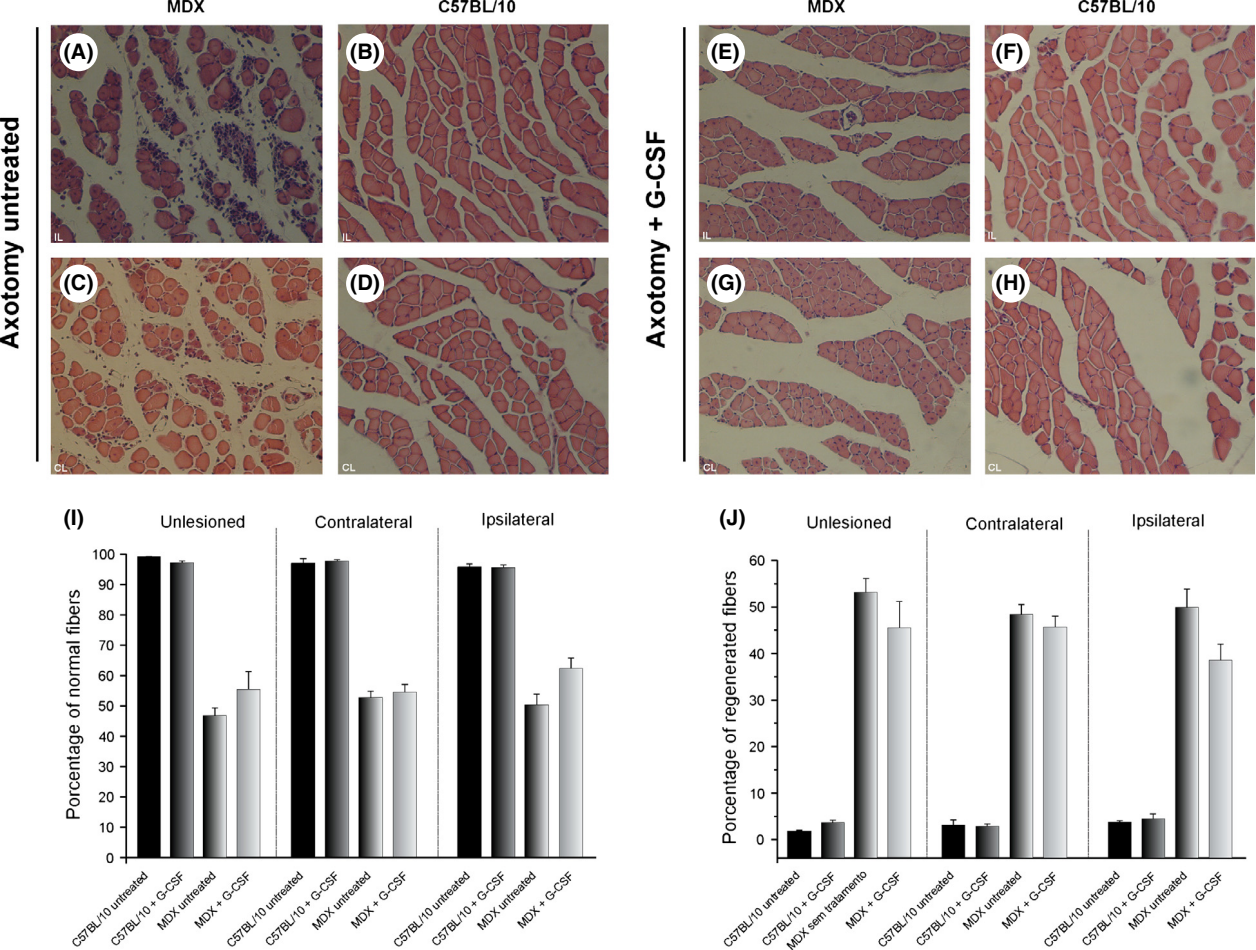
H&E staining. (A and C) The ipsilateral and contralateral soleus muscle of MDX untreated mice subjected to sciatic nerve axotomy. (B and D) the ipsilateral and contralateral soleus muscle in C57BL/10 untreated mice subjected to sciatic nerve axotomy. (E and G) The ipsilateral and contralateral soleus muscle of MDX mice subjected to sciatic nerve axotomy + G-CSF. (F and H) The ipsilateral and contralateral soleus muscle in C57BL/10 mice subjected to sciatic nerve axotomy + G-CSF. (I) The percentage of normal fibers in each strain. (J) The percentage of regenerated fibers in each strain. The normal muscle fibers were identified based on the presence of peripheral nuclei. The regenerated muscle fibers were identified based on the presence of centrally positioned nuclei. *N* = 5 for all experimental groups. Scale bar = 50 *μ*m.” The quantification was performed over the entire nerve area shown in each photo. For I and J, values are the mean ± SEM.

In unlesioned mice, we observed that MDX mice treated with G-CSF showed a higher percentage of normal muscle fibers compared with untreated mice (55% and 46% of normal muscle fibers, respectively, Fig. 6I). Consequently, the percentage of regenerated muscle fibers was lower in the treated mice (45%) compared with untreated mice (54%). In C57BL/10 mice, no differences were observed in the percentage of normal muscle fibers (treatment group, 98%; untreated group, 99%).

Seven days after sciatic nerve axotomy, soleus muscle fiber was quantified. During ipsilateral analysis, we observed a protective effect of the drug on muscle fibers, with MDX + G-CSF group showing that 62% of fibers were unaffected by degeneration. In the MDX untreated group, we observed that 51% of the fibers were not affected by degeneration (Fig. 6I). Thus, 38% of the fibers were regenerated in the MDX + G-CSF group, and 49% were regenerated in the MDX untreated group. In C57BL/10 mice, no differences between untreated and treated groups were observed (95.7 and 95.6% of normal fibers, respectively, Fig. 6J).

## Discussion

Alterations in the spinal cord microenvironment of MDX mice following muscular degeneration/regeneration have been demonstrated previously (Simões and Oliveira 2010, 2012a,b). Using these results, we now use sciatic nerve axotomy to correlate muscle degeneration with the acute effects of peripheral nerve injury along the muscle fibers. Thus, we analyzed the morphology of muscle fibers in the soleus muscle of MDX mice subjected or not subjected to sciatic nerve axotomy. Morphological analyses revealed that 54% of muscle fibers in unlesioned mice were affected by degeneration processes. Seven days after sciatic nerve axotomy, the disconnection between neuronal cell body and its target organ exacerbated the amount of degenerating fibers as well as the motor dysfunction. To protect muscle fibers during the course of muscular dystrophy, we used G-CSF, a drug that shows positive effects on muscle regeneration (Stratos et al. 2007; Naito et al. 2009; Hara et al. 2011). Furthermore, after muscle degeneration G-CSF reduces scar tissue formation, tissue fibrosis, and muscle tissue remodeling, thus enabling the function of the fibers (Okada et al. 2008). Treatment with G-CSF enhances muscle regeneration via the proliferation of satellite cells and by reducing the number of apoptotic cells (Stratos et al. 2007) and increasing the number of myocytes (Stratos et al. 2007; Naito et al. 2009; Hara et al. 2011). In our results, treated MDX mice showed a higher number of normal muscle fibers compared with untreated MDX mice after the first cycle of muscle degeneration, which became more evident after sciatic nerve axotomy, where treated MDX mice showed 62% normal muscle fibers and reduced inflammatory infiltrate, and untreated MDX mice showed 51% fiber preservation and robust inflammatory infiltrate. Thus, we suggest that pretreatment with G-CSF protects muscle fibers during the course of DMD or even after sciatic nerve axotomy, maintains the morphology of the muscle fibers, and enables the fast restructuring of the neuromuscular system. Therefore, we suggest that G-CSF reduces muscle inflammatory processes and protects muscle fibers in the context of the muscular degeneration that occurs in MDX mice.

Additionally, we show positive effects on the skeletal muscular system in MDX mice treated with G-CSF by analyzing the regeneration of axons after peripheral lesions. Here, we use a sciatic nerve crush followed by analysis of motor recovery using the “Catwalk™” system and analysis of nerve morphology in nerves affected by the injury. Our data showed that untreated, placebo, and G-CSF-treated MDX mice exhibited motor deficits compared with C57BL/10 mice even prior to injury (92, 80 and 57% lower, respectively). The G-CSF treatment was more effective during the first days after injury, and it promoted faster recovery compared with the untreated and placebo groups, which was evident during the first 3 days after injury. From the tenth day after injury, the recovery of motor function progressed, and by the end of the experiment, the treated group showed an improved motor function compared with untreated and placebo groups. The effect was most evident in the MDX strain. Our data are consistent with the previous reports in which G-CSF was shown to increase functional recovery in skeletal muscle after muscle injuries (Stratos et al. 2007; Hara et al. 2011). Although associated with functional recovery, G-CSF treatment was insufficient to reverse the motor deficits in MDX mice, which is most likely due to the preexisting muscle fiber degeneration that may cause reduced motor refinement. Therefore, we suggest that the reduction in motor function in MDX mice may be caused by muscle degeneration cycles that directly affect the neuromuscular junctions. The decrease in neuromuscular junctions results in reduced muscle fibers, which are required for moving, and changes motor function. These observations are consistent with our morphological analysis of the soleus muscle, described above. Thus, we suggest that pretreatment in MDX mice during early stages of disease may alleviate disease symptoms.

Morphological analysis of the sciatic nerve was performed using transmission electron microscopy and immunohistochemistry. We observed a reduction in number of regenerated axons in MDX mice compared with C57BL/10 mice after sciatic nerve crush (21% lower in the MDX untreated and MDX placebo groups). However, after treatment with G-CSF, the difference was 1%, showing that G-CSF plays a role in regeneration in the peripheral nervous system.

The thickness of the myelin sheath is a direct indicator of functional recovery of the nerve because it denotes the level of physiological activity of Schwann cells. This parameter has been used in several studies to evaluate the evolution of the axonal regenerative process (Levi and Bunge 1994; Oliveira and Langone 2000; Pierucci et al. 2008). G-CSF treatment reduced the thickness of the myelin sheath and the diameter of the axons in the two strains studied. Moreover, we observed fewer fibers being phagocytosed and greater numbers of axons undergoing remyelination compared with the untreated groups. These data are consistent with functional recovery because a greater number of regenerated axons increase motor units; hence, a greater number of muscle fibers are recruited to execute movement. Thus, we suggest that G-CSF provided a favorable environment for peripheral nerve regeneration and acted beneficially on the activity of Schwann cells in parallel.

**Figure 7:**
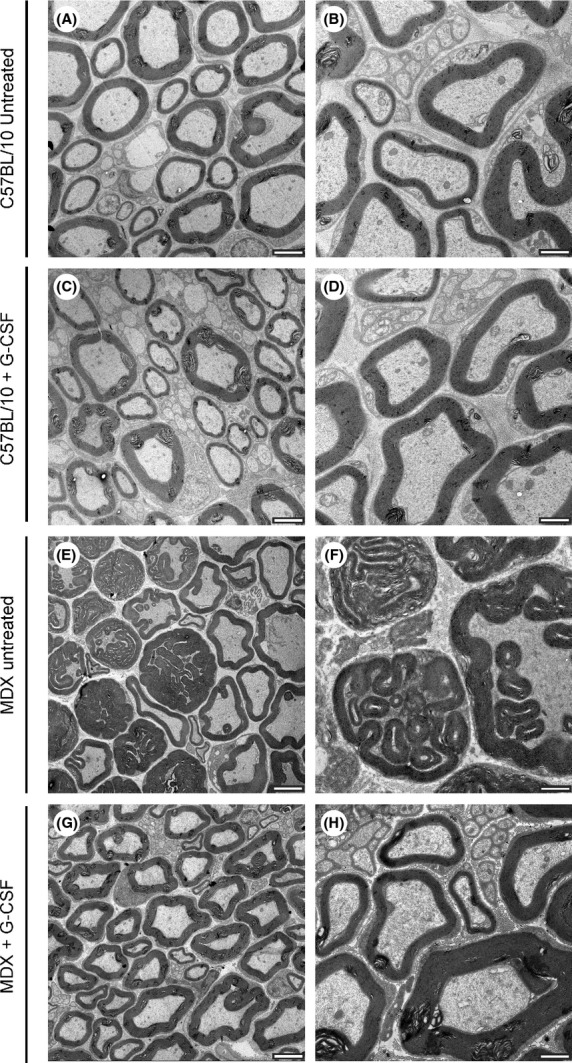
Representative photomicrographs depicting myelination alterations in MDX mice. (A and B) Unlesioned nerves of C57BL/10 mice. (C and D) Unlesioned nerves of C57BL/10 mice treated with G-CSF. (E and F) Unlesioned nerves of MDX mice with malformed myelin sheaths. (G and H) Unlesioned nerves of MDX mice treated with G-CSF. Scale bar = 200 *μ*m for C, E, and G and bar = 1 *μ*m for B, D, F and H.

The “g” ratio (RZG) was established as the ratio between the diameter of the axons and the diameter of the myelin sheath and has been used as a morphometric parameter that indicates the relationship between Schwann cells and axons during regeneration of nerve function (Rushton 1951; Smith and Koles 1970; Waxman 1980). The two treated strains showed reduced “g” ratios, and in the MDX strain, this reduction was greater than the C57BL/10 strain. We suggest that this decrease may be related to the reduction in internodal spaces of myelinated axons due to the proliferation and increased activity of Schwann cells (Ikeda and Oka 2012). Therefore, we assume that Schwann cells of each mouse strain show different metabolic characteristics and that MDX mice show metabolic deficiencies compared with control mice. In this way, these data are consistent with the view that the lower axonal regeneration capacity in MDX mice is directly related to the neural microenvironment. Also, by the ultrastructural observations, it was clear that MDX mice showed malformed myelin sheaths even in the unlesioned subjects (Fig. 7).

Immunohistochemical data showed increased neurofilament and p75^NTR^ immunoreactivity at ipsilateral and contralateral sites to the lesion in untreated, placebo, and treated MDX mice. Based on the p75^NTR^ immunostaining, we believe that the course of disease directly affects Schwann cell activity, which is not sufficient to reestablish homeostasis in the neural microenvironment. Similarly, immunostaining for neurofilament showed an improved reorganization of the regenerated fibers in G-CSF-treated MDX mice, which is consistent with our ultrastructural analyses; this finding again suggests that G-CSF directly increases Schwann cell activity, thereby promoting the fast remyelination of nerve fibers following injury.

Our data suggest that G-CSF is effective in the treatment of peripheral nerve regeneration and that it promotes a favorable microenvironment for axonal regeneration, thereby slowing the progression of Duchenne muscular dystrophy.

## Supporting Information

**Figure S1** Parameters used for morphometric quantification of nerve fibers. Measurements of myelinated fiber diameter (S1-A) and myelinated axons (S1-B) were obtained from the values of their perimeters (P), applying the formula D = P/π. The difference between the myelinated fiber diameter fibers and the myelinated axons provided the thickness of the myelin sheath (S1-C).

**Figure S2** Frequency distribution of the diameter of myelinated fibers in lesioned and unlesioned nerves (contralateral) at 21 days after lesion. Values are presented as the mean ± standard error.

**Figure S3** Frequency distribution of the diameter of myelinated axons in lesioned and unlesioned nerves (contralateral) at 21 days after injury. Values are presented as the mean ± standard error.

**Figure S4** Frequency distribution of the thickness of the myelin sheath in lesioned and unlesioned nerves (contralateral) at 21 days after injury. Values are presented as the mean ± standard error.

**Figure S5** Frequency distribution of the “g” ratio in lesioned and unlesioned nerve (contralateral) at 21 days after injury. Values are presented as the mean ± standard error.

**Figure S6** Electron tomography 3D reconstruction of a malformed myelinated axon found in an unlesioned MDX sciatic nerve. Blue = myelin sheath volume, Gold = axon.
