# Supplementary figures and images for: Granulocyte colony-stimulating factor (G-CSF) positive effects on muscle fiber degeneration and gait recovery after nerve lesion in MDX mice
Source: Brain Behav. 2014 Aug 5;4(5):738–53. doi: 10.1002/brb3.250 (PMC4188366; doi:10.1002/brb3.250)

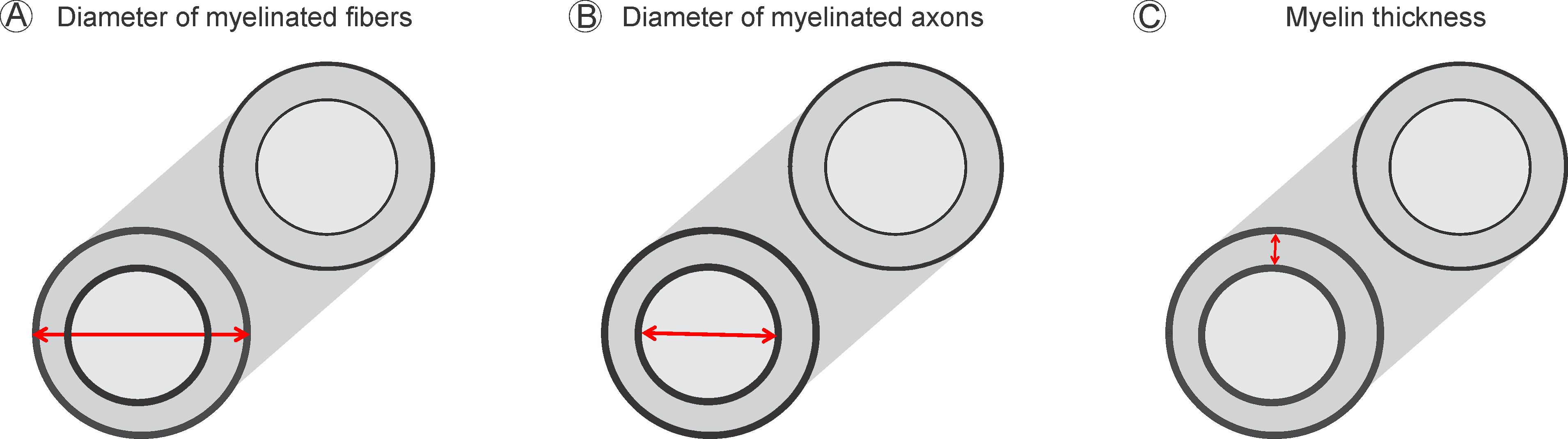

Supplement: Supplementary file 1 — Figure S1 Parameters used for morphometric quantification of nerve fibers. Measurements of myelinated fiber diameter (S1-A) and myelinated axons (S1-B) were obtained from the values of their perimeters (P), applying the formula D = P/π. The difference between the myelinated fiber diameter fibers and the myelinated axons provided the thickness of the myelin sheath (S1-C). [file brb30004-0738-SD1.tif]

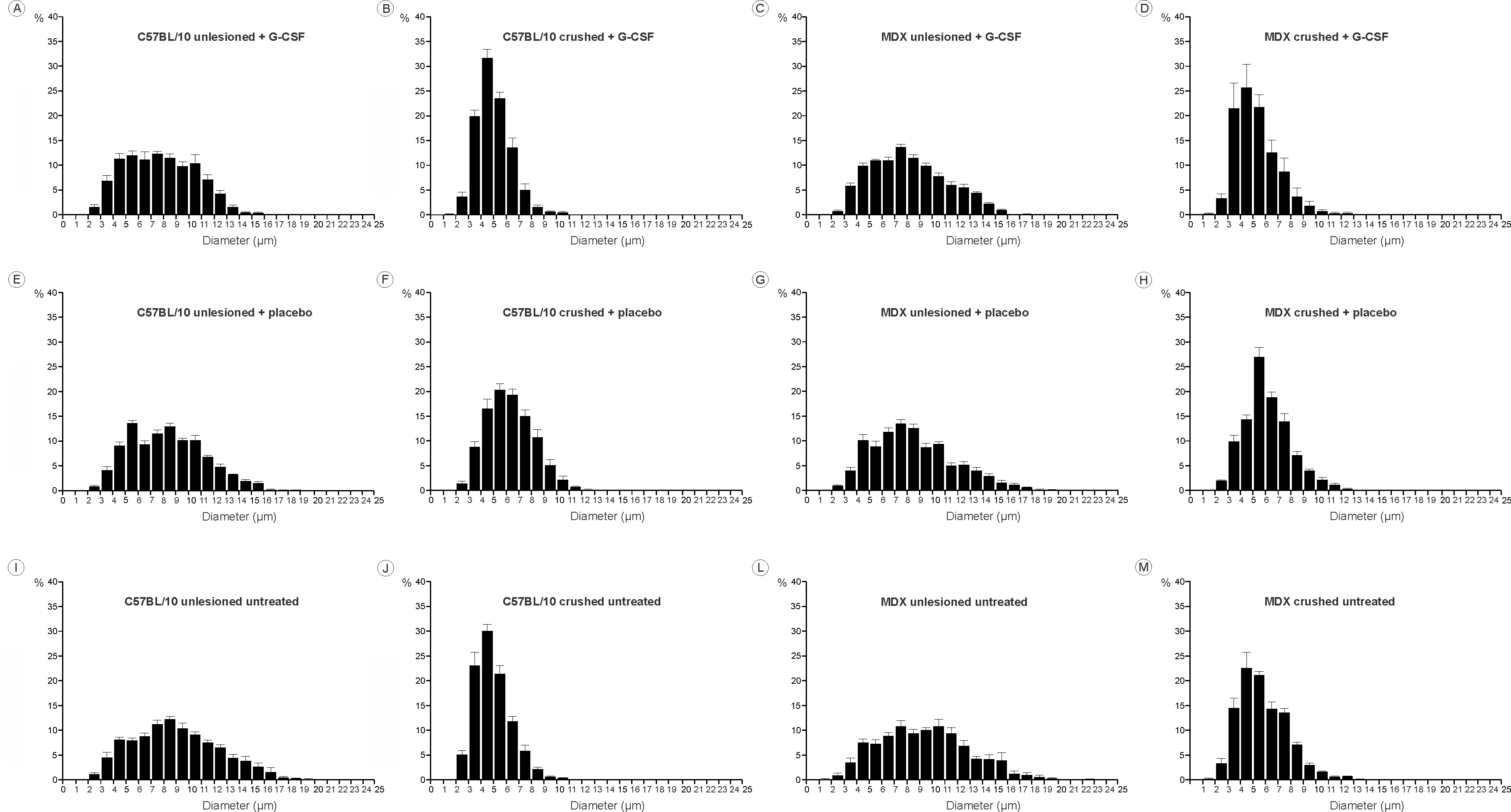

Supplement: Supplementary file 2 — Figure S2 Frequency distribution of the diameter of myelinated fibers in lesioned and unlesioned nerves (contralateral) at 21 days after lesion. Values are presented as the mean ± standard error. [file brb30004-0738-SD2.tif]

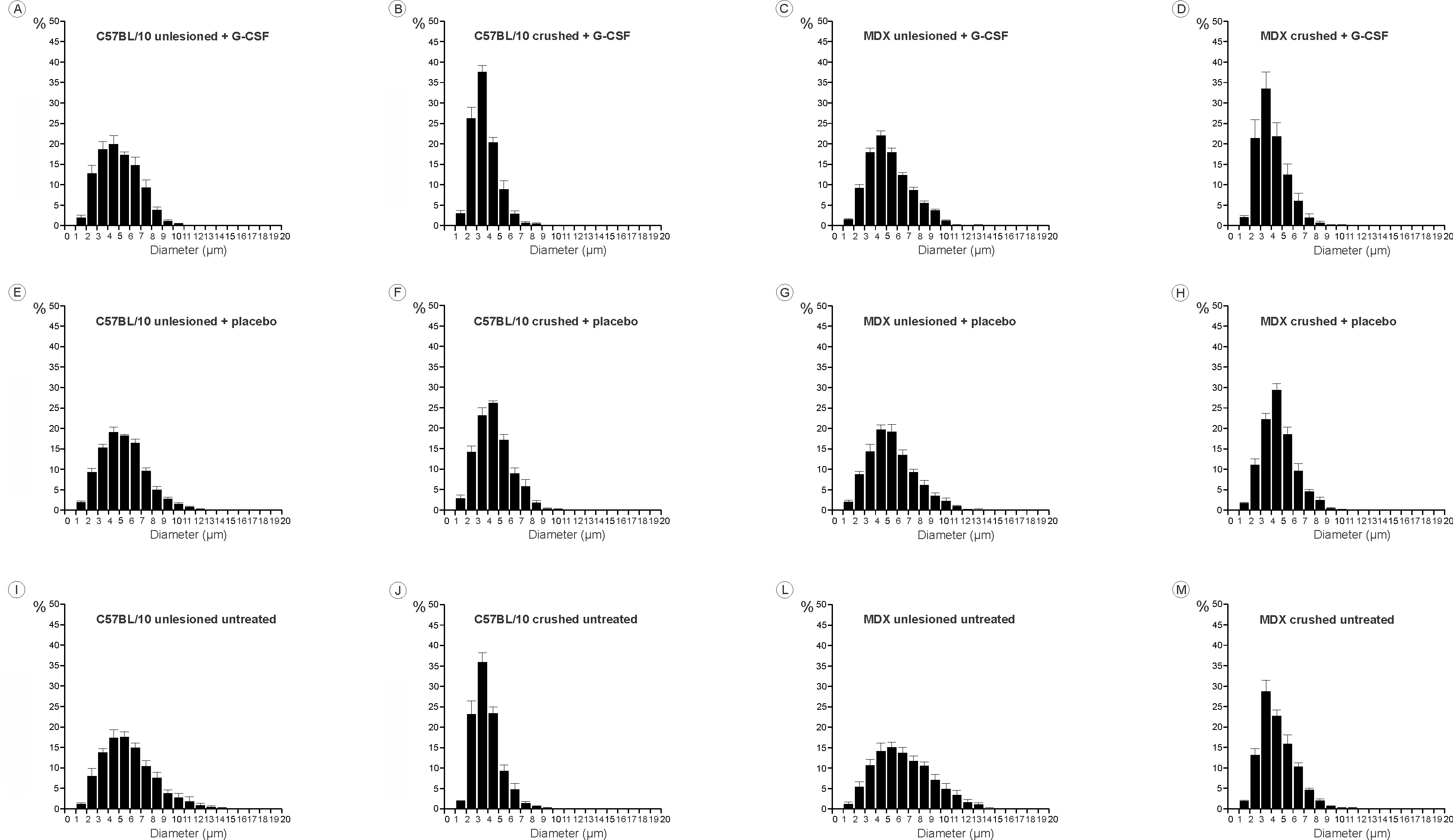

Supplement: Supplementary file 3 — Figure S3 Frequency distribution of the diameter of myelinated axons in lesioned and unlesioned nerves (contralateral) at 21 days after injury. Values are presented as the mean ± standard error. [file brb30004-0738-SD3.tif]

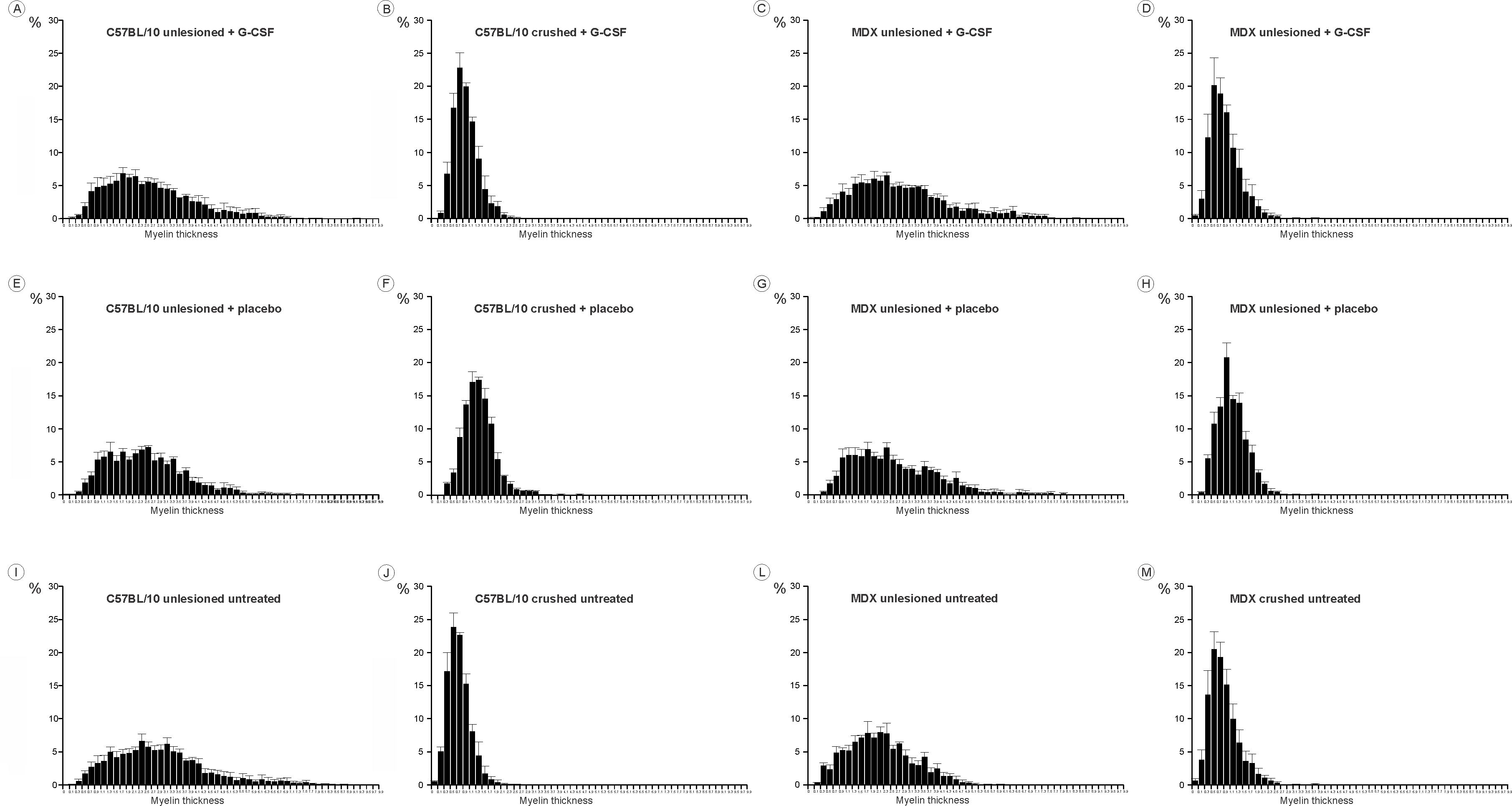

Supplement: Supplementary file 4 — Figure S4 Frequency distribution of the thickness of the myelin sheath in lesioned and unlesioned nerves (contralateral) at 21 days after injury. Values are presented as the mean ± standard error. [file brb30004-0738-SD4.tif]

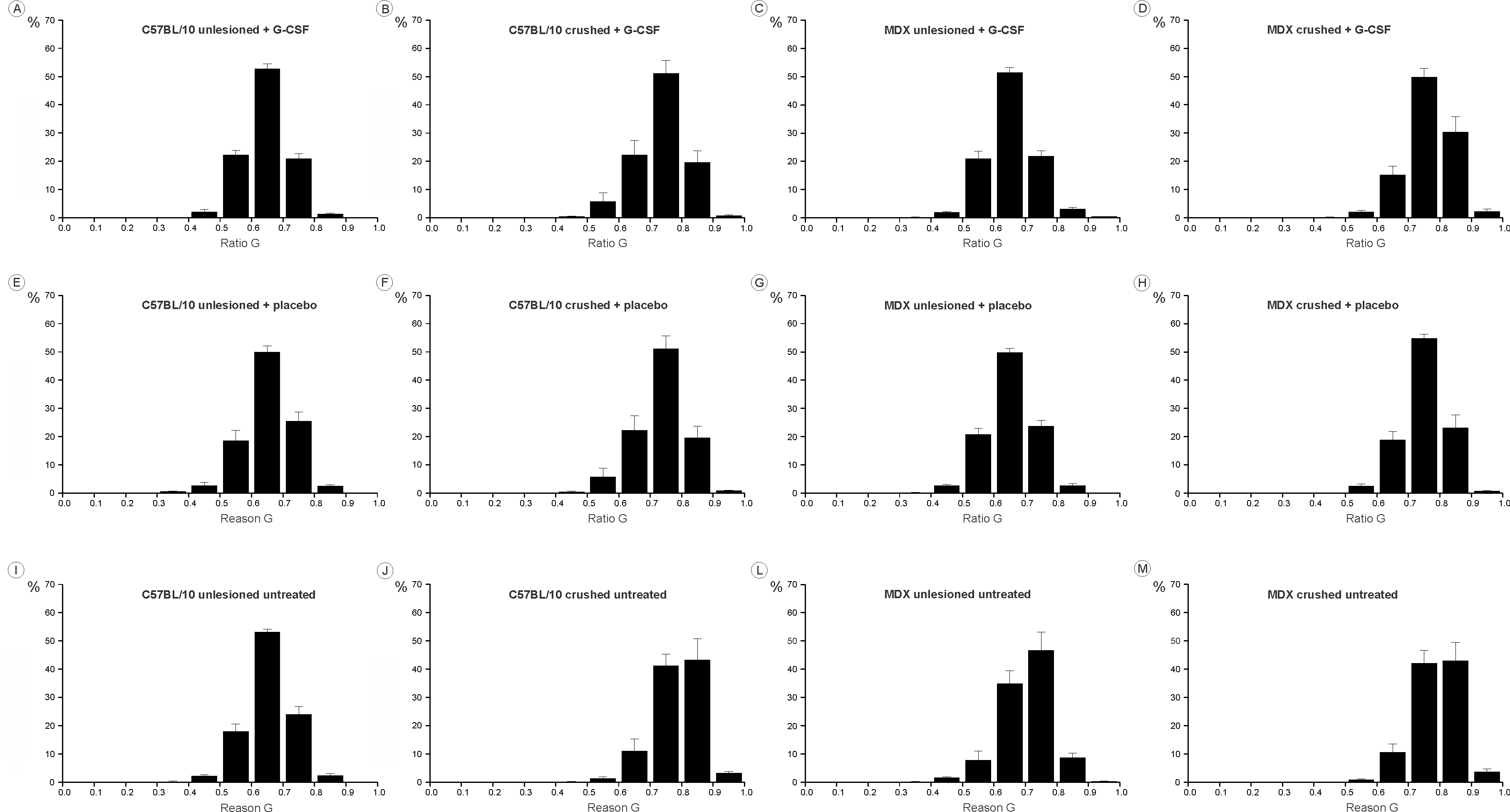

Supplement: Supplementary file 5 — Figure S5 Frequency distribution of the “g” ratio in lesioned and unlesioned nerve (contralateral) at 21 days after injury. Values are presented as the mean ± standard error. [file brb30004-0738-SD5.tif]
