# Supplementary material for: Granulocyte colony-stimulating factor (G-CSF) positive effects on muscle fiber degeneration and gait recovery after nerve lesion in MDX mice
Source: Brain Behav. 2014 Aug 5;4(5):738–53. doi: 10.1002/brb3.250 (PMC4188366; doi:10.1002/brb3.250)
Supplement: Supplementary file 6 — Figure S6 Electron tomography 3D reconstruction of a malformed myelinated axon found in an unlesioned MDX sciatic nerve. Blue = myelin sheath volume, Gold = axon. [file brb30004-0738-SD6.docx]

We have provided a new link for the download of the 3D reconstruction – Figure S6.

<https://www.dropbox.com/sh/7yowuptdiru0l3p/AADBmMow_UCRcYGl6kJazntGa>

**Figure S6:** Electron tomography 3D reconstruction of a malformed myelinated axon found in an unlesioned MDX sciatic nerve. Blue=myelin sheath volume, Gold=axon.
